# Prognostic Relevance of Inflammatory Cytokines Il-6 and TNF-Alpha in Patients with Breast Cancer: A Systematic Review and Meta-Analysis

**DOI:** 10.3390/curroncol32060344

**Published:** 2025-06-11

**Authors:** Jhony A. De La Cruz-Vargas, Henry Gómez, Jesus E. Talavera, Cristhian Gonzales-Rospigliosi, Ariana Alessandra Córdova Salazar, Rafael Pichardo-Rodriguez

**Affiliations:** 1 Institute of Biomedical Sciences Research, Ricardo Palma University, Lima 15039, Peru; hgomezmoreno@gmail.com (H.G.); jesus.talavera@urp.edu.pe (J.E.T.); cristhian.gonzales@urp.edu.pe (C.G.-R.); ariana.cordova@urp.edu.pe (A.A.C.S.); rafael.pichardo@urp.edu.pe (R.P.-R.); 2 Medicina Oncológica, ONCOSALUD-AUNA, Lima 15036, Peru

**Keywords:** breast neoplasms, interleukin-6, tumor necrosis factor-alpha, prognosis

## Abstract

Although cytokines mediate inflammation and inflammation facilitates cancer progression, few studies have evaluated the association between specific cytokines and the prognostic value of breast cancer. Therefore, this study aims to address the following question: What is the prognostic relevance of serum IL-6 and TNF-alpha levels on overall survival and treatment response in women with breast cancer? A systematic review and meta-analysis of cohort studies was conducted. The databases consulted included PubMed/Medline, Web of Science, and EMBASE. A total of 1748 articles were identified, of which 10 were included in the review. A significant association was found between elevated levels of IL-6 and TNF-alpha with poor overall survival and poor treatment response. The meta-analysis showed an HR of 3.74 (95% CI: 1.84–7.6) for elevated IL-6 with high heterogeneity (I^2^: 61%; *p* = 0.07) and an HR of 3.13 (95% CI: 1.57–6.23) for TNF-alpha with low heterogeneity (I^2^: 0%; *p* = 0.9). The overall response rate was 75% (95% CI: 31–100%; I^2^: 92%). In conclusion, IL-6 and TNF-alpha emerge as prognostic inflammatory biomarkers in women with breast cancer and are associated with poor survival and poor treatment response. This study highlights the need to establish an international consensus on cutoff points and standardized determination methods to implement these biomarkers in clinical practice.

## 1. Introduction

Breast cancer is the most frequently diagnosed malignancy and the leading cause of cancer-related mortality among women globally [1,2]. In 2020, 2.3 million new cases and over 600,000 deaths were reported worldwide [2,3]. Additionally, it is estimated that the incidence ranges from 30 to 70 per 100,000 women globally [4].

The prognosis for women with breast cancer has significantly improved worldwide, with a 5-year survival rate of over 99% in localized stages and 32% in advanced stages [5,6]. Despite years of dedicated research on breast cancer patients, it is known that one-third of breast cancer patients may experience relapse, metastasis, and chemotherapy resistance. This underscores the importance of promising biomarkers to clarify the variability in therapeutic response and improve detection and survival [7,8,9].

Specifically, tumor biomarkers are proteins located on the surface of cancer cells that can influence tumor behavior. These biomarkers are valuable in primary, secondary, and tertiary prevention. Their roles include enabling timely diagnosis, facilitating the screening of occult primary cancers, distinguishing between malignant and benign findings, establishing the prognosis of patients diagnosed with cancer, monitoring disease status, predicting tumor response to specific treatments, and detecting recurrences [10]. Depending on the type of information they provide, biomarkers can be classified as prognostic or predictive. If they offer information regarding cancer outcomes (for example, disease progression, recurrence, or overall survival), regardless of the treatment administered, they are considered prognostic biomarkers. Conversely, if they provide information about the effect of a therapeutic intervention—that is, if they are capable of predicting differences in treatment effect between patients with positive biomarkers and those with negative biomarkers—they are categorized as predictive biomarkers. It is crucial to note that some biomarkers provide both predictive and prognostic information [10].

In recent years, significant advances in the prognosis of breast cancer have been made, mainly due to the discovery of various biomarkers and the emergence of targeted therapies [11,12,13]. Molecular markers, such as estrogen receptor (ER), progesterone receptor (PR), human epidermal growth factor receptor 2 (HER2), proliferation marker Ki67, and multigene signatures, which may comprise a set of multiple genes associated with cancer development and progression, are increasingly being used due to their clinical value demonstrated in various randomized clinical trials, and more recently, BRCA1/2 genes, cyclin D1, vascular endothelial growth factor (VEGF), and topoisomerase II (TOPOII) have been extensively studied, and their prognostic value has been established [14,15]. However, these conventional biomarkers are not sufficient to accurately guide treatment decisions and predict prognosis, making the search for new biomarkers a continued focus for researchers [8,9,16,17,18,19,20,21].

IL-6 and TNF-alpha are pro-inflammatory cytokines that have been shown to significantly impact various cancer-related biological processes, including cell proliferation, angiogenesis, invasion, and metastasis [1,22,23]. Previous studies have suggested that elevated levels (IL-6 ≥ 6.81 pg/mL in breast cancer and ≥10 pg/mL in colorectal cancer, and TNF-alpha ≥ 18.93 pg/mL in breast cancer and ≥55 pg/mL in colorectal cancer) of these cytokines are associated with a poorer prognosis in various cancer types, including breast cancer [22,23,24,25,26,27,28,29]. However, evidence regarding their prognostic relevance and influence on treatment response in women with breast cancer remains limited and, in some cases, contradictory, leading to heterogeneity in the results of primary studies concerning clinical outcomes.

An important aspect to highlight is the relevance of combining multiple biomarkers in breast cancer analysis, as this approach can offer a more comprehensive interpretive framework. For instance, elevated IL-6 levels have been associated with reduced survival in patients with HER2-positive breast cancer [30]. Interleukin-6 expression contributes to lapatinib resistance through maintenance of the stemness property in HER2-positive breast cancer cells. The combination of biomarkers, including IL-6 and ER, facilitates the identification of breast cancer subtypes with distinct characteristics and biological behaviors [31]. Notably, IL-6 overexpression promotes resistance to anti-HER2 therapy in HER2-positive breast cancer models [1].

This study, through a systematic review and meta-analysis, examined the outcomes of 19 articles that classified breast cancer based on the presence or absence of specific biomarkers (ER, PR, HER2, IL-6, TNF-alpha). The scientific literature presents a variety of suggested treatments for breast cancer, including surgery (as primary therapy for early-stage cancer), chemotherapy (both adjuvant and anthracycline based), hormonal therapy (for hormone-sensitive cancers), neoadjuvant chemotherapy, and evaluation of adjuvant chemotherapy guided by genetic recurrence scores. However, several studies primarily focus on investigating the prognostic and diagnostic roles of cytokines, such as IL-6 and TNF-α, as well as genetic markers, like HER2 polymorphism, without providing exhaustive details on the specific treatment regimens received by patients. In these cases, the emphasis is placed on how these biomarkers are associated with disease progression, overall treatment response, or survival outcomes.

The aim of this study was to evaluate the prognostic relevance of serum IL-6 and TNF-alpha levels on survival and treatment response in women with breast cancer.

## 2. Materials and Methods

### 2.1. Registry

A systematic review (SR) with a meta-analysis of observational studies was conducted. The PRISMA statement [32] was used as a guideline for reporting systematic reviews and meta-analyses. This review was registered in the International Prospective Register of Systematic Reviews (PROSPERO) with ID CRD42024569952.

### 2.2. Eligibility Criteria

Studies involving research on the prognostic value of inflammatory cytokines IL-6 and TNF-alpha in patients with breast cancer were included. Records in Spanish and English were considered, provided they were available in full-text versions. Excluded were letters to the editor, case reports, case–control studies, cross-sectional studies, descriptive studies, editorials, commentaries, press articles, clinical trials, reviews, and/or conference abstracts.

### 2.3. Search Strategy and Study Selection

The search strategy included the following databases: PubMed/Medline, Web of Science, and EMBASE. The key terms used were IL-6 and TNF-alpha. The search strategy for each database is available in Supplementary File S1. The search was conducted between 10 June and 10 July 2024.

All documents containing the combination of the following descriptors were included: (’breast cancer’ OR ’breast gland neoplasm’ OR ’breast malignancies’ OR ’cancer in the mammary gland’) AND (’interleukin 6’ OR ’IL-6’ OR ’TNF-alpha’ OR ’TNF-alpha’ OR ’recombinant tumor necrosis factor alpha’ OR ’tumor necrosis factor alpha’ OR ’tumor necrosis factor alpha’) AND (’overall survival’ OR ’progression-free survival’ OR ’survival’ OR ’complete response’ OR ’complete response rate’ OR ’survival rate’ OR ’survival curve’ OR ’survival probability’ OR ’treatment response’ OR ’therapeutic response’ OR ’therapy response’). The search was conducted independently and blinded by two authors (A.C. and E.T.). No restrictions were applied regarding publication date to ensure the inclusion of all available information, regardless of the laboratory quantification methodology used.

Rayyan software was used for study selection (https://rayyan.qcri.org) (accessed on 29 June 2024), with the objective of storing the articles found in the search from each database. Two researchers (J.E.T. and A.C.S.) reviewed the titles and abstracts to select potential studies for inclusion. Finally, the research team independently evaluated the full-text version of each potential study to determine eligibility (the table of excluded articles is available in Supplementary File S2). Disagreements were resolved by the decision of a third reviewer (R.P.-R.).

### 2.4. Data Extraction

Data extraction from the selected articles was performed using a data collection sheet in Microsoft Excel 2016. The following information was extracted from each selected article: author, year, country, study type, sample size, cytokine measurements (IL-6 and TNF-alpha), cutoff points, type of breast cancer, follow-up duration, outcomes, and treatment response.

The exposure variables were IL-6 and TNF-alpha, both measured in two categories: elevated or not elevated, according to the cutoff points reported in the studies. The outcome variables included survival (overall survival, progression-free survival, and disease-free survival) and treatment response (complete response, partial response, stable disease, or disease progression).

### 2.5. Risk of Bias Assessment

To assess the quality of the selected studies, the Newcastle–Ottawa risk of bias tool, modified in the review by Modesti et al. [33], was used. This tool is widely used in systematic reviews and meta-analyses to assess the quality of observational studies, particularly cohort studies. For cohort studies, it evaluates three key areas to determine methodological quality: selection, comparability, and outcomes.

In the selection domain, it assesses the representativeness of the cohorts and distinguishes between exposed and non-exposed groups. For comparability, it accounts for confounding variables. In outcomes, it analyzes the accuracy of the outcomes, the follow-up period, and participant retention to ensure reliability.

This tool was chosen due to its ability to evaluate multiple validity elements in observational studies. It provides a standardized and replicable classification of quality, facilitating the comparison of heterogeneous studies and contributing to the reduction of biases in data synthesis.

This process was conducted by two independent researchers (J.E.T. and A.C.S.), and in case of disagreement, a third researcher (R.P.-R.) made the final decision. The criteria used for the evaluation are specified in Table 1.

### 2.6. Statistical Analysis

Measure of association.

Hazard ratio adjusted.

### 2.7. Response to Treatment

- Overall response rate (ORR)

$$\left(\frac{\mathrm{Number}\;\mathrm{of}\;\mathrm{patients}\;\mathrm{with}\;\mathrm{complete}\;\mathrm{response}+\mathrm{Number}\;\mathrm{of}\;\mathrm{patients}\;\mathrm{with}\;\mathrm{partial}\;\mathrm{response}\;}{\mathrm{Total}\;\mathrm{Number}\;\mathrm{of}\;\mathrm{Treated}\;\mathrm{Patients}\;}\right)\times 100\%$$

- Complete response rate (CRR)

$$\left(\frac{\mathrm{Number}\;\mathrm{of}\;\mathrm{patients}\;\mathrm{with}\;\mathrm{complete}\;\mathrm{response}\;}{\mathrm{Total}\;\mathrm{Number}\;\mathrm{of}\;\mathrm{Treated}\;\mathrm{Patients}\;}\right)\times 100\%$$

- Partial response rate (PRR)

$$\left(\frac{\mathrm{Number}\;\mathrm{of}\;\mathrm{patients}\;\mathrm{with}\;\mathrm{partial}\;\mathrm{response}}{\mathrm{Total}\;\mathrm{Number}\;\mathrm{of}\;\mathrm{Treated}\;\mathrm{Patients}\;}\right)\times 100\%$$

### 2.8. Qualitative Synthesis

An evaluation of all collected articles was conducted to provide an understanding of the characteristics of shared decision-making support tools. Clinical and methodological characteristics were described (e.g., included studies, sample size, inclusion and exclusion criteria), as well as the strengths and weaknesses of all included studies. The analysis also addressed how study design or execution might bias the results, the relationship between study characteristics, and the reported outcomes.

#### 2.8.1. Quantitative Synthesis

Heterogeneity was assessed using Cochran’s Q test (*p*-value < 0.10 was considered statistically significant) and I^2^ (>75%: high heterogeneity; 25–50%: moderate heterogeneity; <25%: low heterogeneity) [52]. For the quantitative synthesis, a meta-analysis of adjusted hazard ratios was performed using the inverse variance method with a random-effects model, following the DerSimonian and Laird approach [53]. Additionally, a fixed-effects model was applied [53]. For the synthesis of overall, complete, and partial response rates, a meta-analysis of proportions was conducted using the double arcsine transformation method [54]. A publication bias analysis represented in a forest plot was performed [55]. The analysis was performed using R, with the ’metagen’ and ’metaprop’ functions (version 4.1.2; R Project for Statistical Computing) and key R packages, such as ’meta’.

#### 2.8.2. Additional Analyses

A sensitivity analysis (influence analysis) using the Leave-One-Out method was performed to assess the influence of each study on heterogeneity. A meta-regression analysis was also conducted to evaluate potential factors contributing to heterogeneity.

## 3. Results

### 3.1. Eligible Studies

**Figure 1:**
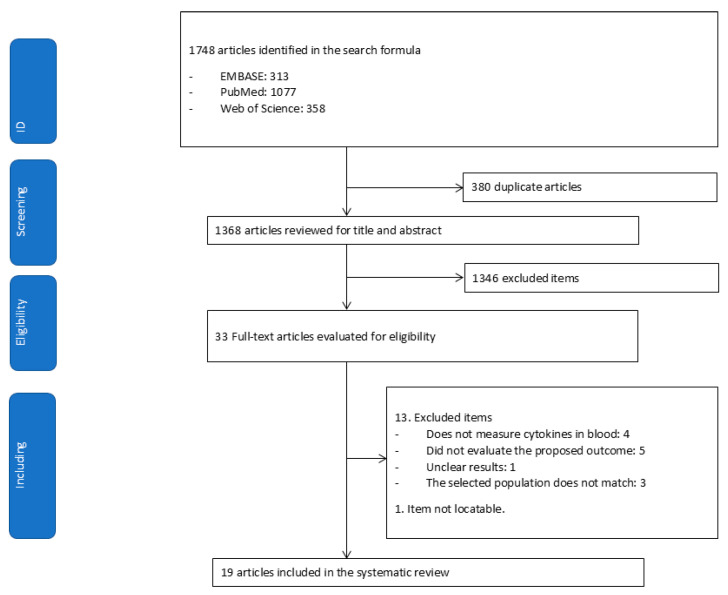
Flowchart for extracting relevant articles from the literature.

A total of 1748 articles were identified through database searches. After removing duplicates, 1368 articles were screened, of which 1346 were excluded and 33 were assessed in full text. Of those thirty-three, thirteen were excluded, and one could not be retrieved. Ultimately, 19 articles were included in the systematic review (Figure 1).

### 3.2. Study Characteristics

Table 2 presents the main characteristics of the studies. Of the 19 studies [23,34,35,36,37,38,39,40,41,42,43,44,45,46,47,48,49,50,51], the included studies (n = 2505 women with breast cancer) had sample sizes ranging from 12 to 532 women per study. The studies were published between 1999 and 2022. All included studies were cohort studies. Cytokines were evaluated in seventeen studies (85%) that used an Enzyme-Linked Immunosorbent Assay (ELISA) in serum or plasma; one study (5%) (52) used gene expression analysis in co-culture systems, and the remaining 10% used a Chemiluminescence Immunoassay (CLIA) [34,50].

Fifty-five percent of the studies did not report a cutoff point for IL-6; among those that did, the median cutoff for elevated IL-6 was 6.8 pg/mL. Similarly, 50% of the studies [34,35,36,38,41,42] did not report a cutoff point for TNF-alpha; among those that did, the median cutoff for elevated TNF-alpha was 18.9 pg/mL.

Twenty-five percent of the studies did not indicate a follow-up period, while the remaining seventy-five percent reported a median follow-up time of 36 months (range: during treatment to 12.6 years). Seven studies reported data on treatment response, with Berberoglu et al. [36] being one of the few to report complete response rates, which was 15% (n = 3). For further details, see Table 2.

### 3.3. Risk of Bias Assessment

The methodological quality of the 19 included studies was assessed using the Newcastle–Ottawa Scale (NOS) adapted for longitudinal designs. Overall, the majority of studies (n = 16; 84%) were classified as having a high risk of bias, while three studies (16%) were rated as low risk 

The domains of exposure assessment and outcome evaluation were adequately addressed in all studies (100%), indicating consistent and reliable measurement of cytokine levels and clinical endpoints. However, other domains showed substantial limitations. Specifically, only five out of nineteen studies (26%) met the criterion for representativeness of the exposed cohort, and none included an unexposed comparison group, which may limit external validity and introduce potential selection bias.

In terms of confounding control, only six studies (32%) reported adequate statistical or methodological adjustment for important prognostic factors. The remaining 13 studies (68%) did not clearly describe or apply confounding control, suggesting a moderate to high risk of internal bias in the reported associations.

With regard to follow up, twelve studies (63%) reported an adequate duration of at least five years, while seven studies (37%) either did not reach this threshold or failed to report the length of follow up. However, fifteen studies (79%) were deemed to have adequate follow-up completeness (i.e., low attrition or well-documented losses), whereas four studies (21%) did not report sufficient information on cohort retention (Table 1).

### 3.4. Meta-Analysis for IL-6 and Breast Cancer

**Figure 2:**
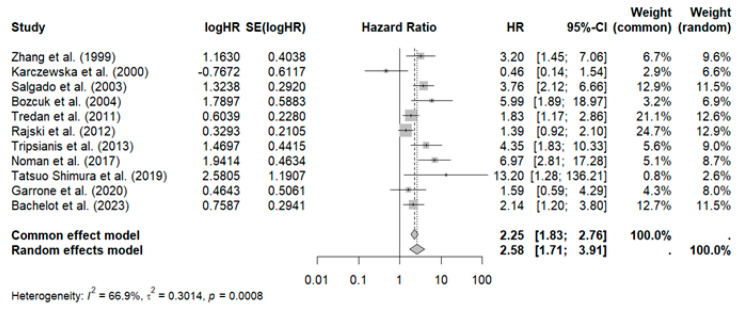
Forest plot of overall survival in patients with breast cancer on IL-6 levels [35,38,40,43,44,45,47,48,49,50,51].

Eleven studies with 1062 patients reported an association between elevated IL-6 and poor overall survival (HR: 2.25, 95% CI: 1.83–2.76) with moderate heterogeneity (I^2^: 66%; *p* < 0.001), as shown in Figure 2.

**Figure 3:**
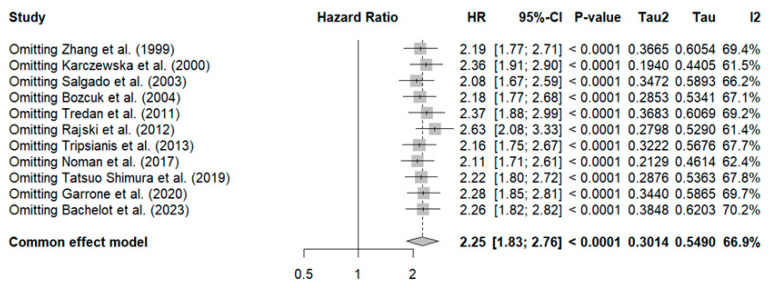
Sensitivity analysis of overall survival in breast cancer patients for IL-6 [35,38,40,43,44,45,47,48,49,50,51].

Sensitivity analysis revealed that omitting the study by Rajski et al. reduced heterogeneity (HR: 2.63, 95% CI: 2.08–3.33, I2: 61.4%), as shown in Figure 3.

Additionally, to identify the source of heterogeneity, a meta-regression was conducted to analyze the impact of sample size, which was found to have no significant effect (*p* = 0.3). See Supplementary File S3 for further details. Publication bias is evident in the Funnel plot, showing asymmetry with a predominance on the right side (studies with a higher estimated risk). See Supplementary File S4.

### 3.5. Meta-Analysis for TNF-Alpha and Breast Cancer

Three studies with 254 patients reported no association between elevated TNF-alpha and poor overall survival (HR: 2.06, 95% CI: 0.98–4.31) with high heterogeneity (I^2^: 55%; *p* = 0.11).

### 3.6. Meta-Analysis for Treatment Response

**Figure 4:**
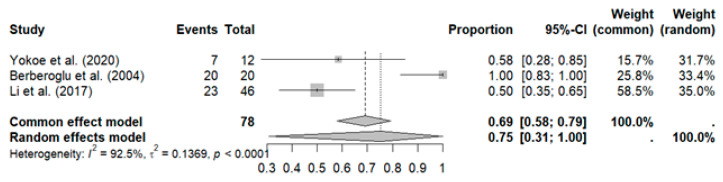
Meta-analysis for treatment response [34,36,41].

Three studies with a total of 78 patients reported a high overall response rate (ORR): pooled ORR (ORRpooled: 75%, 95% CI: 31–100%) with high heterogeneity (I^2^: 92%; *p* < 0.01) (see Figure 4).

**Figure 5:**
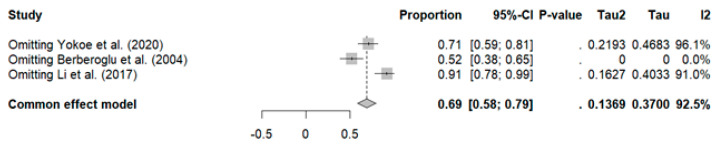
Sensitivity analysis of overall rate response in breast cancer patients [34,36,41].

Sensitivity analysis revealed that excluding the study by Berberoglu et al. reduced heterogeneity to zero (ORRpooled: 52%, 95% CI: 38–65%, I^2^: 0%), along with a decrease in the pooled ORR, as shown in Figure 5.

It was not possible to perform a subgroup analysis of the ORR stratified by elevated or low levels of IL-6 and TNF-α, as the included studies did not report these data in sufficient detail, limiting the corresponding analysis.

## 4. Discussion

### 4.1. Main Results

In this study, the objective was to evaluate the prognostic relevance of serum levels of IL-6 and TNF-alpha in survival and treatment response in women with breast cancer. The findings suggest that inflammation plays a dual role in cancer, potentially both promoting and inhibiting tumor progression, which influences treatment response and survival [24]. Cytokines such as IL-6 and TNF-alpha have been consistently associated with lower overall survival and unfavorable response in women with breast cancer [23]. This relationship has been established in both experimental and clinical studies [1,22] and has also been observed in other types of cancer [25,26,27]. The findings of this study are consistent with the literature but emphasize the need to interpret the results with caution due to the observed heterogeneity.

Response rates were not reported by all studies; however, a pooled response rate (TRGpooled) of 75% was estimated, which is comparable to the response rates achieved with new treatments [56].

### 4.2. Heterogeneity

A significant source of heterogeneity was the absence of a specific cutoff point for IL-6 and TNF-alpha, which made it difficult to establish an association with overall survival (OS). Due to the scarcity of studies, it was not possible to conduct a subgroup analysis to determine whether values above or below a particular point present a stronger or weaker association with OS. Additionally, it was noted that 55% of the studies did not report a cutoff point for IL-6 and TNF-alpha, highlighting the importance of establishing an international consensus. In the meantime, each region should define its own cutoff point based on the characteristics of its population.

Another point to discuss is the laboratory methodology for determining serum cytokine levels. Seventeen studies used ELISA [57], and two used chemiluminescence (ECLIA) [58]. According to the literature, ELISA should currently be preferred due to its more suitable linear range and performance in immunological profiling. However, ECLIA represents an emerging technology with potential, though future studies are needed to evaluate its clinical performance in large sample cohorts [59]. It is crucial to highlight as a limitation of this study that, due to the different methodologies and automated equipment used, it is challenging to perform a precise quantitative synthesis of the results.

With the available evidence, we propose preliminary cutoff points for distinguishing high and low cytokine levels: 6.8 pg/mL for IL-6 and 18.9 pg/mL for TNF-α. However, these values require validation in well-designed prospective studies that include adequate sample sizes and representative control groups, as well as standardized analytical methodologies, to confirm their clinical utility and prognostic accuracy.

### 4.3. Biological Plausibility

During chronic inflammation induced by cancer, tissues responding to IL-6 gradually become resistant, correlating with elevated IL-6 levels [57]. This cytokine can trigger uncontrolled inflammatory responses, leading to chronic inflammation and even carcinoma [1]. Furthermore, the hyperactivation of the IL-6/JAK/STAT3 signaling pathway can suppress antitumor immune responses in the tumor microenvironment, allowing cancer to persist [1]. This could explain why patients with elevated IL-6 levels exhibit worse overall survival compared to those with lower levels.

TNF-α, another cytokine implicated in breast cancer, plays a crucial role in three aspects. First, a correlation has been found between TNF-α levels at the tumor site or in the plasma/serum of breast cancer patients, their clinical status, and outcomes. Second, TNF-α plays a significant role in signaling in both estrogen receptor-positive and estrogen receptor-negative breast cancer cells. Third, TNF-α is involved in epithelial–mesenchymal transition and breast cancer cell metastasis, contributing to the development of drug resistance [22].

### 4.4. Implications and Practical Perspectives

The use of IL-6 and TNF-alpha as prognostic biomarkers could enhance the personalization of treatment in breast cancer patients by tailoring it according to the risk associated with overall survival and potential therapeutic response. However, the absence of a defined cutoff point hinders their immediate clinical application, making it necessary to establish regional thresholds until a global consensus is reached.

As an additional perspective for future research, it is important to highlight that in preclinical studies, the administration of tocilizumab, a monoclonal antibody inhibitor of IL-6, has demonstrated a significant delay in tumor progression compared to control groups [60]. These findings have spurred the development of clinical trials aimed at evaluating the potential of tocilizumab as an adjuvant therapy in breast cancer patients [61,62].

The initial results of a recent clinical trial confirmed the safety of tocilizumab in combination with standard treatments. However, conclusive data on its efficacy in terms of survival or tumor response have not yet been reported [63]. The available evidence is currently limited and fragmented. Additional results are awaited to clarify the clinical impact of IL-6 inhibition on improving therapeutic outcomes for breast cancer patients. No preclinical or clinical evidence was found regarding the efficacy of TNF-alpha antibodies in breast cancer patients.

### 4.5. Limitations

The main limitation of this study lies in the scarcity of research and the absence of an internationally accepted threshold for IL-6 and TNF-alpha. The lack of sufficient data to conduct subgroup analyses estimating the pooled response rate (TRGpooled) based on high or low levels of IL-6 and TNF-alpha represents a major limitation. This restriction prevents an adequate evaluation of the potential impact of these cytokine levels on treatment response, further complicating the assessment of the potential clinical benefit that could arise from their inhibition through specific therapeutic interventions.

## 5. Conclusions

IL-6 and TNF-alpha are emerging as prognostic biomarkers of poor survival in breast cancer. It is imperative to move toward an international consensus to define a cutoff point and standardize the measurement methodology. Further studies are needed to determine the impact of these cytokines on the clinical management of patients and therapeutic response.

## Figures and Tables

**Table 1:** Quality assessment criteria for included studies using the Newcastle–Ottawa scale (NOS) adapted for longitudinal studies.

Author, Years	Selection				Comparability		Result		Score	Final Judgment
	Representativeness of the Sample ^1^	Selection of the Unexposed Cohort ^2^	Determination of Exposure ^3^	Outcome of Interest NOT Present at Baseline ^4^	The Study Controls for the Most Important Factor or Additional Factors ^5^	Outcome Assessment ^6^	Adequate Follow-Up Time ^7^	Adequacy of Cohort Follow Up ^8^		
Yokoe et al., 2000 [34]	-	-	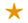	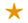	-	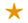	-	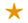	4	High Risk
Bachelot et al., 2003 [35]	-	-	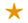	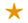	-	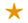	-	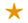	4	High Risk
Berberoglu et al., 2004 [36]	-	-	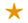	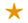	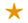	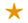	-	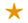	5	High Risk
Papadopoulou et al., 2010 [37]	-	-	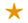	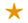	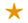	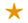	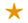	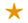	6	High Risk
Noman et al., 2017 [38]	-	-	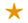	-	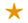	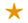	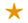	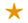	5	High Risk
Fuksiewicz et al., 2010 [39]	-	-	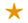	-	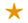	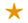	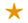	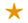	5	High Risk
Tripsianis et al., 2013 [40]	-	-	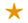	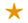	-	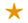	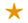	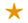	5	High Risk
Tripsianis et al., 2014 [23]	-	-	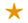	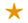	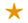	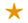	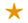	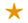	6	High Risk
Li et al., 2017 [41]	-	-	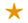	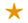	-	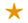	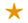	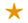	5	High Risk
Sparano et al., 2022 [42]	-	-	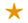	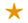	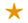	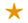	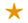	-	5	High Risk
Trédan et al., 2011 [43]	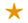	-	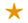	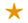	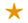	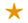	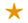	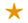	7	Low Risk
Bozcuk et al., 2004 [44]	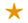	-	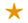	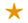	-	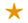	-	-	5	High Risk
Salgado et al., 2003 [45]	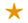	-	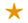	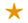	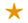	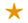	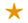	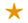	7	Low Risk
Cho et al., 2013 [46]	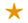	-	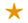	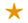	-	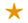	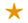	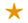	6	High Risk
Rajski et al., 2012 [47]	-	-	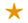	-	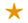	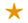	-	-	3	High Risk
Shimura et al., 2019 [48]	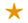	-	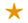	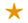	-	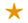	-	-	5	High Risk
Garrone et al., 2020 [49]	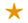	-	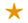	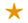	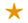	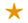	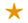	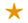	7	Low Risk
Zhang and Adachi, 1999 [50]	-	-	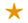	-	-	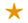	-	-	3	High Risk
Karczewska et al., 2000 [51]	-	-	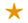	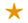	-	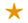	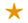	-	4	High Risk

^1^ Representativeness of the exposed cohort: One star was assigned to studies with random sampling or census. ^2^ Selection of the unexposed cohort: One star was assigned to studies where the unexposed cohort came from the same community as the exposed cohort. ^3^ Determination of exposure: The way in which the dependent variable was measured was clearly explained. ^4^ Outcome of interest not present at baseline: A star was assigned to studies that showed that the outcome of interest was not present at baseline. ^5^ Study controls for the most important factor or additional factors: An adjustment, either methodological or statistical, was made for the most important confounding variable or for other confounding variables. ^6^ Evaluation of outcomes: If the study explicitly stated how outcomes were measured using a reliable assessment, a star was given. ^7^ Adequate follow-up time: If the study followed patients well, for approximately 5 years, a star was assigned. ^8^ Adequacy of cohort follow up: A star was assigned if all subjects in the study completed follow up or if missing subjects did not introduce bias.

**Table 2:** Characteristics and results of the included studies on the association between blood cytokine levels and breast cancer.

Author, Year	Country	Study Type	Sample	Measurement of IL-6 and TNF-α	Cutoff Point for IL-6 and TNF-α	Type of Breast Cancer *	Follow-Up Time	**Outcome**	**Response to Treatment**
Zhang and Adachi (1999) [50]	Japan	Observational	46 women with metastatic breast cancer	IL-6: CL-EIA, TNF-α	4 pg/mL	Metastatic	Up to 32 months	Overall survival, response to treatment	High IL-6 = lower OSand response
Karczewska et al. (2000) [51]	Poland	Retrospective observational	75 women with breast carcinoma	IL-6/IL-6R/gp130 (ARNm in tissue)	Positive vs. negative expression	Early and advanced	61 months (median)	Overall survival, Progression- free survival	Not specified
Yokoe et al., 2000 [34]	Japan	Cohort study	12 women with recurrent breast cancer, with an average age of 51 years	IL-6: CLIA chemiluminescent immunoassay (blood)	Does not report	ER+ (7), PR (3)	70 months	Response to treatment (partial or null)	Partial response (n = 7)
Bachelot et al., 2003 [35]	France	Cohort study	87 women with metastatic breast cancer (median 54)	IL-6: serum enzyme immunoassay kit	IL-6: <13 and 55 pg/mL ≥13 and 55 pg/mL	ER+ (58) ER− (23) It is not known (6)	Does not indicate	Overall survival	Did not report
Salgado et al. (2003) [45]	Belgium	Prospective observational study	96 women with previously untreated metastatic cancer	IL-6 serum (ELISA)	6.6 pg/mL (median)	metastatic	12 months (median)	Overall survival	IL-6 high = worse prognosis
Berberoglu et al., 2004 [36]	Turkey	Prospective cohort study	20 women with locally advanced breast cancer (median 45 years) and 12 healthy women as controls (median 43 years)	TNF-α: ELISA (serum)	Does not report	ER+ (13), ER− (7), PR+ (11), PR− (9)	Does not indicate	Response to treatment (complete or partial)	Partial response (n = 17) and complete response (n = 3)
Bozcuk et al. (2004) [44]	Turkey	Prospective	43 women with metastatic breast cancer	IL-6 and TNF-α serum	Non-specific (continuous)	Metastatic	Does not indicate	Overall survival, Progression- free survival	Association with progression
Trédan et al. (2011) [43]	France	Prospective observational	299 women with advanced or metastatic cancer	IL-6 serum (immunoassay)	Does not report	Advanced/metastatic	12 months (median)	Overall survival	Did not report
Papadopoulou et al., 2010 [37]	Greece	Prospective cohort study (case–control)	56 women with primary breast cancer (median 64 years) and 45 healthy women (median 59 years)	TNF-α: ELISA (serum)	TNF-α: <11 pg/mL ≥11 pg/mL	ER+ (37), ER− (19), PR+ (25), PR− (31) HER-2− (25) HER-2+ (31)	30 months (median)	Overall survival	Did not report
Fuksiewicz et al., 2010 [39]	Poland	Prospective cohort study	210 women with breast cancer (median age 54 years)	TNF-α and IL-6: ELISA (serum)	IL6 (2.4 pg/mL) TNF-α (4.4 pg/mL)	Does not report	9 years (median)	Overall survival and disease-free survival	Did not report
Rajski et al. (2012) [47]	Switzerland	Experimental + clinical in silico	295 tumors (co-culture and in silico)	IL-6 expression induced in co-culture	High vs. low gene expression	Early (bone metastasis)	12.6 years (median)	Time to bone metastasis,	Not applicable (bioinformatics)
Tripsianis et al., 2013 [40]	Greece	Prospective cohort study	130 women with primary breast cancer (median age 65 years)	TNF-α and IL-6: ELISA (serum)	IL-6: <7.12 pg/mL ≥7.12 pg/mL TNF-α: <18.80 pg/mL ≥18.80 pg/mL	ER+ (81), PR+ (62)	31 months (median)	Overall survival	Did not report
Cho et al. (2013) [46]	Korea	Prospective	240 women with breast cancer	IL-6, IL-8, serum leptin	By subgroups	HER2–, ER+ /PR+	6 years	Progression-free survival (recurrence)	Associated with recurrence
Tripsianis et al., 2014 [23]	Greece	Prospective cohort study	112 women with primary breast cancer (median 65 years) and 45 healthy women	IL-6 and TNF-α: ELISA (serum)	IL-6: <6.81 pg/mL TNF-α: <18.93 pg/mL	ER+ (78), ER- (34), PR+ (48), PR− (64) HER-2− (51) HER-2+ (61)	30 months (median)	Survival	Did not report
Li et al., 2017 [41]	China	Retrospective cohort study	46 women with breast cáncer	IL-6: ELISA (serum)	Does not report	Luminal, (30) HER-2 (10), Basal (6)	5 years	Response to treatment (complete, partial, null) Overall survival	Partial response + Complete response (n = 23)
Noman et al., 2017 [38]	Bangladesh	Prospective cohort study	110 patients (65 with untreated progressive metastatic breast cancer) and 30 healthy women	IL-6: ELISA (serum)	Does not report	Does not report	3 years	and event-free survival	Did not report
Shimura et al. (2019) [48]	Japan	Retrospective observational	55 women with invasive breast cancer	Serum IL-6 and PCR	IL-6 ≥ 10 pg/mL; PCR ≥ 0.12 mg/dl	Invasive	Not specified	Overall survival, Recurrence- free survival	Not reported
Garrone et al. (2020) [49]	Italy	Prospective translational	41 patients with metastatic cancer (treated with eribulin)	IL-6, TNF-α, IL-8, IL-10, IL-21 (plasma)	By median	Metastatic	During treatment	Overall survival, Progression-free survival	Evaluation by progression
Sparano et al., 2022 [42]	USA	Prospective retrospective cohort study	532 women	IL-6 and TNF-α: V-human cytokine plex, (serum)	Does not report	ER and PR− (180) ER and PR+ (318)	Approx. 5 years	Remote recurrence	Did not report

* Breast cancer type: ER+ (estrogen receptor positive), ER− (estrogen receptor negative), PR+ (progesterone receptor positive), PR− (progesterone receptor negative), HER-2 (human epidermal growth factor receptor-type 2).

## Data Availability

This review was registered in the International Prospective Register of Systematic Reviews (PROSPERO) with ID CRD42024569952. The search strategy for each database is available in Supplementary Materials.

## Supplementary Materials

The following supporting information can be downloaded at: https://www.mdpi.com/article/10.3390/curroncol32060344/s1, File S1: Search strategy; File S2: List of articles not included; File S3: Meta-regression; File S4: Publication bias.

## Abbreviations

The following abbreviations are used in this manuscript:

ER	Estrogen receptor
PR	Progesterone receptor
HER2	Human epidermal growth factor receptor 2
VEGF	Vascular endothelial growth factor
TOPOII	Topoisomerase II
SR	Systematic review
EIA	Enzyme immunoassay
CLIA	Chemiluminescence immunoassay
NOS	Newcastle–Ottawa scale
OS	Overall survival
ORR	Overall response rate
CRR	Complete response rate
PRT	Partial response rate
