# Supplementary material for: Prognostic Relevance of Inflammatory Cytokines Il-6 and TNF-Alpha in Patients with Breast Cancer: A Systematic Review and Meta-Analysis
Source: Curr Oncol. 2025 Jun 11;32(6):344. doi: 10.3390/curroncol32060344 (PMC12192186; doi:10.3390/curroncol32060344)
Supplement: Supplementary file 1 [file curroncol-32-00344-s001.zip › File S1.pdf]

File S1. SEARCH STRATEGY

Search terms used in various combinations include "IL-6," "TNF-alpha," "breast cancer or tumor or carcinoma," and "prognosis or outcome or survival"

- ✓ Search strategy for each search engine

PUBMED

('breast cancer' OR 'ca breast' OR 'breast cancer' OR 'breast gland cancer' OR 'breast gland neoplasm' OR 'breast malignancies' OR 'breast malignancy' OR 'breast tumor malignant' OR 'cancer in the mammary gland' OR 'cancer of the breast' OR 'cancer of the mammary gland' OR 'cancer, breast' OR 'malignancies of the breast' OR 'malignancy of the breast' OR 'malignant breast neoplasm' OR 'malignant breast tumor' OR 'malignant neoplasm of the breast' OR 'malignant tumor of the breast' OR 'mamma cancer' OR 'mammary cancer' OR 'mammary gland cancer' OR 'mammary gland malignancy' OR 'mammary malignancies' OR 'mammary malignancy') AND ('interleukin 6' OR '26 k protein' OR 'b cell stimulatory factor 2' OR 'b cell stimulating factor 2' OR 'b lymphocyte stimulating factor 2' OR 'beta 2 interferon' OR 'beta2 interferon' OR 'bsf 2' OR 'bsf2' OR 'hepatocyte stimulating factor' OR 'il 6' OR 'interferon beta 2' OR 'interferon beta2' OR 'interleukin 6' OR 'interleukin b' OR 'interleukin hp1' OR 'interleukin-6' OR 'liver cell stimulating factor' OR 'plasmacytoma growth factor' OR 'protein 26k' OR 'tumor necrosis factor' OR 'tnf alfa' OR 'tnf alpha' OR 'cachectin' OR 'cachetin' OR 'human recombinant tumour necrosis factor alpha' OR 'mhr 24' OR 'recombinant tumour necrosis factor alpha' OR 'tissue necrosis factor' OR 'tumor necrosis factor' OR 'tumor necrosis factor alfa' OR 'tumor necrosis factor alpha' OR 'tumor necrosis factor-alpha' OR 'tumor necrosis factors' OR 'tumor necrosis serum' OR 'tumour necrosis factor' OR 'tumour necrosis factor alfa' OR 'tumour necrosis factor alpha' OR 'tumour necrosis factor-alpha' OR 'tumour necrosis factors' OR 'tumour necrosis serum') AND ('overall survival' OR 'overall survival' OR 'progression free survival' OR 'pfs (progression free survival)' OR 'progression free' OR 'progression free survival' OR 'progression-free survival' OR 'survival' OR 'survival' OR 'complete response' OR 'complete response rate' OR 'survival rate' OR 'rate, survival' OR 'survival curve' OR 'survival probability' OR 'survival rate' OR 'treatment response' OR 'therapeutic response' OR 'therapy response' OR 'treatment response') AND ('observational study' OR 'non experimental studies' OR 'non experimental study' OR 'nonexperimental studies' OR 'nonexperimental study' OR 'observation studies' OR 'observation study' OR 'observational studies' OR 'observational studies as topic' OR 'observational study' OR 'observational study as topic' OR 'cohort analysis'/exp OR 'analysis, cohort' OR 'cohort analysis' OR 'cohort fertility' OR 'cohort life cycle' OR 'cohort studies' OR 'cohort study' OR 'fertility, cohort' OR 'case control study')

WEB OF SCIENCE

TS=(('breast cancer' OR 'ca breast' OR 'breast gland cancer' OR 'breast gland neoplasm' OR 'breast malignancies' OR 'breast malignancy' OR 'breast tumor malignant' OR 'cancer in the mammary gland' OR 'cancer of the breast' OR 'cancer of the mammary gland' OR 'cancer, breast' OR 'malignancies of the breast' OR 'malignancy of the breast' OR 'malignant breast neoplasm' OR 'malignant breast tumor' OR 'malignant neoplasm of the breast' OR 'malignant tumor of the breast' OR 'mamma cancer' OR 'mammary cancer' OR 'mammary gland cancer' OR 'mammary gland malignancy' OR 'mammary malignancies' OR 'mammary malignancy') AND ('interleukin 6' OR '26 k protein' OR 'b cell

stimulatory factor 2' OR 'b cell stimulating factor 2' OR 'b lymphocyte stimulating factor 2' OR 'beta 2 interferon' OR 'beta2 interferon' OR 'bsf 2' OR 'bsf2' OR 'hepatocyte stimulating factor' OR 'il 6' OR 'interferon beta 2' OR 'interferon beta2' OR 'interleukin 6' OR 'interleukin b' OR 'interleukin hp1' OR 'interleukin-6' OR 'liver cell stimulating factor' OR 'plasmacytoma growth factor' OR 'protein 26k' OR 'tumor necrosis factor' OR 'tnf alfa' OR 'tnf alpha' OR 'cachectin' OR 'cachetin' OR 'human recombinant tumour necrosis factor alpha' OR 'mhr 24' OR 'recombinant tumour necrosis factor alpha' OR 'tissue necrosis factor' OR 'tumor necrosis factor' OR 'tumor necrosis factor alfa' OR 'tumor necrosis factor alpha' OR 'tumor necrosis factor-alpha' OR 'tumor necrosis factors' OR 'tumor necrosis serum' OR 'tumour necrosis factor' OR 'tumour necrosis factor alfa' OR 'tumour necrosis factor alpha' OR 'tumour necrosis factor-alpha' OR 'tumour necrosis factors' OR 'tumour necrosis serum') AND ('overall survival' OR 'progression free survival' OR 'pfs (progression free survival)' OR 'progression free' OR 'progression-free survival' OR 'survival' OR 'complete response' OR 'complete response rate' OR 'survival rate' OR 'survival curve' OR 'survival probability' OR 'treatment response' OR 'therapeutic response' OR 'therapy response') AND ('observational study' OR 'non experimental studies' OR 'non experimental study' OR 'nonexperimental studies' OR 'nonexperimental study' OR 'observation studies' OR 'observation study' OR 'observational studies' OR 'observational studies as topic' OR 'observational study' OR 'observational study as topic' OR 'cohort analysis' OR 'cohort study' OR 'case control study'))

EMBASE

('breast cancer'/exp OR 'ca breast' OR 'breast cancer' OR 'breast gland cancer' OR 'breast gland neoplasm' OR 'breast malignancies' OR 'breast malignancy' OR 'breast tumor malignant' OR 'cancer in the mammary gland' OR 'cancer of the breast' OR 'cancer of the mammary gland' OR 'cancer, breast' OR 'malignancies of the breast' OR 'malignancy of the breast' OR 'malignant breast neoplasm' OR 'malignant breast tumor' OR 'malignant neoplasm of the breast' OR 'malignant tumor of the breast' OR 'mamma cancer' OR 'mammary cancer' OR 'mammary gland cancer' OR 'mammary gland malignancy' OR 'mammary malignancies' OR 'mammary malignancy') AND ('interleukin 6'/exp OR '26 k protein' OR 'b cell stimulatory factor 2' OR 'b cell stimulating factor 2' OR 'b lymphocyte stimulating factor 2' OR 'beta 2 interferon' OR 'beta2 interferon' OR 'bsf 2' OR 'bsf2' OR 'hepatocyte stimulating factor' OR 'il 6' OR 'interferon beta 2' OR 'interferon beta2' OR 'interleukin 6' OR 'interleukin b' OR 'interleukin hp1' OR 'interleukin-6' OR 'liver cell stimulating factor' OR 'plasmacytoma growth factor' OR 'protein 26k' OR 'tumor necrosis factor'/exp OR 'tnf alfa' OR 'tnf alpha' OR 'cachectin' OR 'cachetin' OR 'human recombinant tumour necrosis factor alpha' OR 'mhr 24' OR 'recombinant tumour necrosis factor alpha' OR 'tissue necrosis factor' OR 'tumor necrosis factor' OR 'tumor necrosis factor alfa' OR 'tumor necrosis factor alpha' OR 'tumor necrosis factor-alpha' OR 'tumor necrosis factors' OR 'tumor necrosis serum' OR 'tumour necrosis factor' OR 'tumour necrosis factor alfa' OR 'tumour necrosis factor alpha' OR 'tumour necrosis factor-alpha' OR 'tumour necrosis factors' OR 'tumour necrosis serum') AND ('overall survival'/exp OR 'overall survival' OR 'progression free survival'/exp OR 'pfs (progression free survival)' OR 'progression free' OR 'progression free survival' OR 'progression-free survival' OR 'survival'/exp OR 'survival' OR 'complete response'/exp OR 'complete response rate'/exp OR 'survival rate'/exp OR 'rate, survival' OR 'survival curve' OR 'survival probability' OR 'survival rate' OR 'treatment response'/exp OR 'therapeutic response' OR 'therapy response' OR 'treatment response') AND ('observational study'/exp OR 'non experimental studies' OR 'non experimental study' OR 'nonexperimental studies'

OR 'nonexperimental study' OR 'observation studies' OR 'observation study' OR 'observational studies' OR 'observational studies as topic' OR 'observational study' OR 'observational study as topic' OR 'cohort analysis'/exp OR 'analysis, cohort' OR 'cohort analysis' OR 'cohort fertility' OR 'cohort life cycle' OR 'cohort studies' OR 'cohort study' OR 'fertility, cohort' OR 'case control study'/exp)

- ✓ Results of the search strategy according to each database

**Database**	**Results**
PUBMED	1077
WEB OF SCIENCE	358
EMBASE	313
