# Supplementary material for: Prognostic Relevance of Inflammatory Cytokines Il-6 and TNF-Alpha in Patients with Breast Cancer: A Systematic Review and Meta-Analysis
Source: Curr Oncol. 2025 Jun 11;32(6):344. doi: 10.3390/curroncol32060344 (PMC12192186; doi:10.3390/curroncol32060344)
Supplement: Supplementary file 1 [file curroncol-32-00344-s001.zip › File S2.pdf]

File S2. LIST OF ARTICLES NOT INCLUDED

Code	Study	Reason for exclusion
1	Circulating Cytokines in Metastatic Breast Cancer Patients Select Different Prognostic Groups and Patients Who Might Benefit from Treatment beyond Progression	Measuring cytokines with another method
2	Tissue polypeptide antigen & interleukin-6: Are their serum levels a predictor for response to chemotherapy in breast cancer?	Does not include survival or response to treatment
3	Associations of circulating C-reactive protein and interleukin-6 with survival in women with and without cancer: Findings from the British Women's Heart and Health Study	Population with different types of cancer
4	Association between serum cytokines and progression of breast cancer in Chinese population.	Measuring cytokines with another method
5	Inflammatory Cytokines and Comorbidity Development in Breast Cancer Survivors Versus Noncancer Controls: Evidence for Accelerated Aging?	No incluye supervivencia ni respuesta al tratamiento
6	Changes of cytokines and thyroid function in patients with recurrent breast cancer	Result off topic
7	TNF-alpha, IL-6 and their soluble receptor serum levels and secretion by neutrophils in cancer patients.	Population with different types of cancer
8	Relationship satisfaction predicts lower stress and inflammation in breast cancer survivors: A longitudinal study of within-person and between-person effects.	Does not include survival or response to treatment
9	Elevated level of peripheral CD8(+) CD28(-) T lymphocytes are an independent predictor of progression-free survival in patients with metastatic breast cancer during the course of chemotherapy.	Result off topic
10	Host genetic variants in the interleukin-6 promoter predict poor outcome in patients with estrogen receptor-positive, node-positive breast cancer.	Study with an off-topic focus
11	Clinicopathological and prognostic significance of serum cytokine levels in breast cancer.	Measuring cytokines by another method
12	Validation of prognostic scores for survival in cancer patients beyond first-line therapy.	Population with different types of cancer
