# Supplementary material for: Prognostic Relevance of Inflammatory Cytokines Il-6 and TNF-Alpha in Patients with Breast Cancer: A Systematic Review and Meta-Analysis
Source: Curr Oncol. 2025 Jun 11;32(6):344. doi: 10.3390/curroncol32060344 (PMC12192186; doi:10.3390/curroncol32060344)
Supplement: Supplementary file 1 [file curroncol-32-00344-s001.zip › File S3.pdf]

File S3. META- REGRESSION

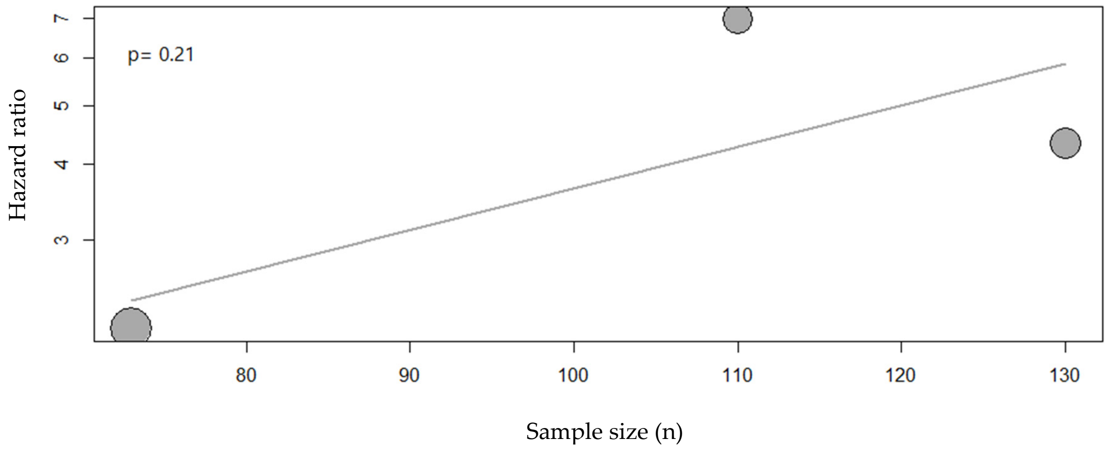

File S3. Absence of influence of sample size on heterogeneity according to meta-regression analysis in Overall Survival for IL-6.
