# Supplementary material for: Prognostic Relevance of Inflammatory Cytokines Il-6 and TNF-Alpha in Patients with Breast Cancer: A Systematic Review and Meta-Analysis
Source: Curr Oncol. 2025 Jun 11;32(6):344. doi: 10.3390/curroncol32060344 (PMC12192186; doi:10.3390/curroncol32060344)
Supplement: Supplementary file 1 [file curroncol-32-00344-s001.zip › File S4.pdf]

File S4 PUBLICATION BIAS

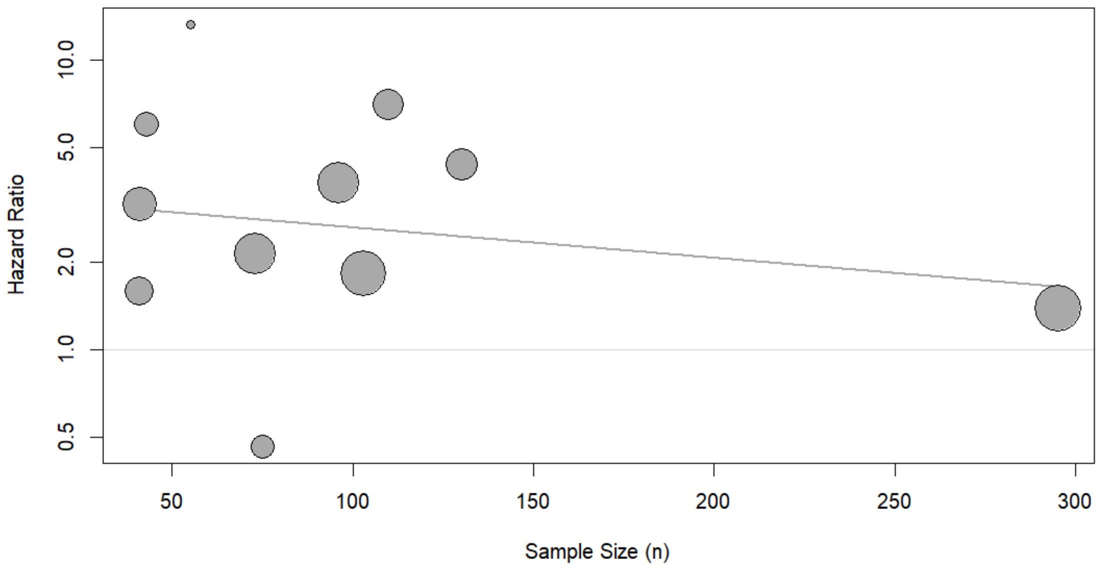

File S4. Publication bias is evident in the Funnel plot, showing asymmetry with a predomi-nance on the right side (studies with a higher estimated risk).
